# Design and Simulation of Low-Threshold Miniaturized Single-Mode Nanowire Lasers Combined with a Photonic Crystal Microcavity and Asymmetric Distributed-Bragg-Reflector Mirrors

**DOI:** 10.3390/nano10122344

**Published:** 2020-11-26

**Authors:** Chao Wu, Wei Wei, Xueguang Yuan, Yangan Zhang, Xin Yan, Xia Zhang

**Affiliations:** 1 State Key Laboratory of Information Photonics and Optical Communications, Beijing University of Posts and Telecommunications, Beijing 100876, China; wuchao920073513@bupt.edu.cn (C.W.); yuanxg@bupt.edu.cn (X.Y.); xzhang@bupt.edu.cn (X.Z.); 2 School of Mechanical and Electric Engineering, Guangzhou University, Guangzhou 510006, China; wei@gzhu.edu.cn; 3 Photonics Research Centre, Department of Electronic and Information Engineering, The Hong Kong Polytechnic University, Hung Hom, Kowloon 999077, Hong Kong, China

**Keywords:** nanowire lasers, photonic crystal, distribute-Bragg-reflector, single mode, multi-wavelength laser, InGaAs

## Abstract

A low-threshold miniaturized single-mode nanowire laser operating at telecommunication wavelengths was proposed and simulated. The device was constructed by combining a single InGaAs nanowire with a photonic crystal microcavity and asymmetric distributed-Bragg-reflector mirrors. The mode characteristics and threshold properties were calculated using the three-dimensional finite-different time-domain method. Due to the effective subwavelength confinement and strong optical feedback, provided by the photonic crystal microcavity, and distributed-Bragg-reflector mirrors, respectively, the confinement factor, end-facet reflectivity, and quality factor significantly improved. A lowest threshold of ~80 cm^−1^ and ultra-small cut-off radius of ~40 nm are obtained, reduced by 67%, and 70%, respectively, compared with a traditional nanowire laser. In addition, due to the photonic band gap effect, single-mode lasing is achieved with a high side-mode suppression ratio of >12 dB. By placing several identical nanowires in the photonic crystal with different lattice constants, an on-chip laser array is realized, which is promising in wavelength division multiplexing applications. This work may pave the way for the development of low-threshold miniaturized nanolasers and low-consumption high-density photonic integrated circuits.

## 1. Introduction

Low-threshold miniaturized semiconductor lasers are critical devices for low-consumption high-density photonic integrated circuits. In the past decades, different types of structures have been developed to achieve microscale lasers, including micro-sized vertical cavity surface emitting lasers (VCSELs) [1,2], microdisks [3,4,5,6], microtubes [7,8,9,10,11], and so on. To further improve the power consumption and integration density, nanoscale semiconductor lasers have attracted increasing attention in recent years. Semiconductor nanowire (NW) is a quasi-one-dimensional material with a nanoscale footprint and microscale length. Due to the miniaturized size, high refractive index, and natural Fabry-Perot (F-P) cavity configuration, semiconductor NWs have shown great potential in low-consumption nanoscale lasers. So far, NW lasers with different materials have been demonstrated, which have covered the ultraviolet, visible, and short wave near-infrared wavelengths [11,12,13,14,15,16,17,18]. However, the traditional photonic NW lasers are restricted in optical mode size and physical device dimension caused by the optical diffraction limit. As the diameter decreases, the mode confinement becomes weaker and threshold increases. In addition, due to the NWs’ small size compared with the wavelength, the mirror loss is large and the reflectivity of end facets is poor for small-diameter NWs, which further increases the threshold.

To further reduce the dimension and consumption of NW lasers, different structures have been developed, in which surface plasmon polariton (SPP) lasers have gained much attention due to the strong subwavelength confinement. Although, SPP NW lasers operating at the ultraviolet, visible, and short wave near-infrared wavelengths have been demonstrated or proposed, the large loss of noble metals at long wave near-infrared wavelengths makes it difficult to operate at telecommunication wavelengths [19,20,21]. Photonic crystal (PhC) microcavity is another promising structure to achieve subwavelength confinement over a broad wavelength range. In particular, by engineering the lattice constant of the PhC, the lasing wavelength can be easily tuned, enabling the realization of NW laser array for on-chip wavelength division multiplexing (WDM) applications. So far, PhC-based NW lasers have rarely been reported [22,23,24,25,26,27,28,29,30]. In addition, for both the SPP and PhC NW lasers, the poor end-facet reflectivity leads to a high threshold, which makes lasing difficult at small diameters even the mode is well confined. Hence, to achieve low-threshold miniaturized NW lasers, the confinement factor and end-facet reflectivity should be promoted at the same time. 

In this work, a PhC microcavity and asymmetric distributed-Bragg-reflector (DBR) mirrors are introduced to improve the confinement factor and end-facet reflectivity of single NWs for achieving low-threshold miniaturized NW lasers. An InGaAs NW emitting around 1550 nm is placed in the line defect waveguide of a silicon-on-insulator (SOI)-based PhC for subwavelength confinement. Asymmetric DBRs composed of silicon/air and silicon/silica pairs are placed on two ends of NW to enhance the optical feedback. The simulation results show that the PhC microcavity and DBR mirrors provide effective subwavelength confinement, and strong optical feedback, respectively, leading to significantly enhanced confinement factor, end-facet reflectivity, and quality factor. A lowest threshold of ~80 cm^−1^ and ultra-small cut-off radius of ~40 nm are obtained, reduced by 67%, and 70%, respectively, compared with a traditional NW laser. In addition, due to the photonic band gap effect, single-mode lasing is achieved with a high side-mode suppression ratio of >12 dB. By adjusting the lattice constant of the PhC, the lasing wavelength could be effectively tuned, enabling the realization of on-chip laser array for wavelength division multiplexing applications (WDM) with identical NWs. This work may provide effective guidance for the realization of low-threshold miniaturized nanolasers and high-density photonic integrated circuits.

## 2. Methods

**Figure 1:**
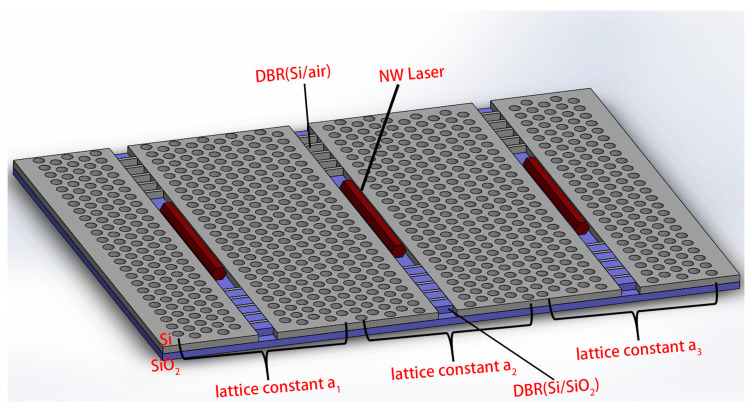
The schematic diagram of the multi-wavelength NW lasers combined with a PhC microcavity and asymmetric DBRs on the SOI chip.

The structure of the multi-wavelength In_0.52_Ga_0.48_As [31] NW lasers combined with a PhC microcavity and asymmetric DBRs on the SOI chip is depicted in Figure 1. A triangular- lattice hole silicon PhC slab with a thickness of 200 nm was fabricated on top of the SOI chip, where the lattice constant a_1_, a_2_, and a_3_ were 445 nm, 453 nm, and 461 nm, respectively, and the radius of holes was 120 nm. The line defect waveguide in the PhC was 500 nm in width and 200 nm in depth. Identical NWs with a length of 4 μm were placed in the line defect waveguide. To enhance the optical feedback, asymmetric DBRs, were placed on two ends of each NW, where the higher-reflectivity DBR was composed of silicon/air pairs, while the lower-reflectivity DBR was composed of silicon/silica pairs. The lasing wavelength could be tuned simply just by changing the lattice constant of the PhC. Electromagnetic field distribution in this work was calculated by Lumerical FDTD solutions with perfectly matched layers boundary conditions, which absorbed the fields leaving the simulated region in order to prevent reflections. Here, the mode source with a center wavelength of 1550 nm was used, corresponding to the band-edge emission of In_0.52_Ga_0.48_As at room temperature.

**Figure 2:**
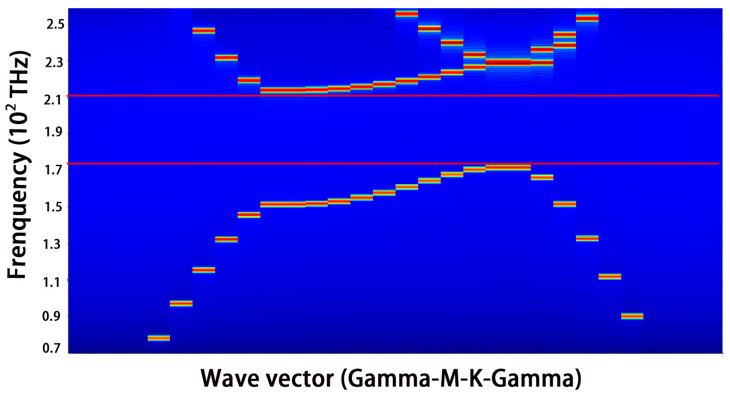
The photonic band gap of the complete PhC.

It is well-established that a subwavelength NW is too thin to form an efficient optical cavity by itself due to the optical diffraction limit. As the radius decreases, the confinement factor sharply reduces. By introducing a silicon PhC, the light leaked from the NW could be re-localized into the NW gain medium attributed to the photonic bandgap, which significantly improves the confinement factor of transverse modes [32,33]. The photonic band gap of the PhC with a lattice constant of 453 nm is shown in Figure 2. The frequency of the lasing designed is around 193.4 THZ, which is observed in the middle of the photonic band gap of the PhC. The line defect waveguide is expected to create one conduction band close to 1550 nm, which not only raises the confinement factor, but also enables single-mode lasing by selecting the required longitudinal mode.

**Figure 3:**
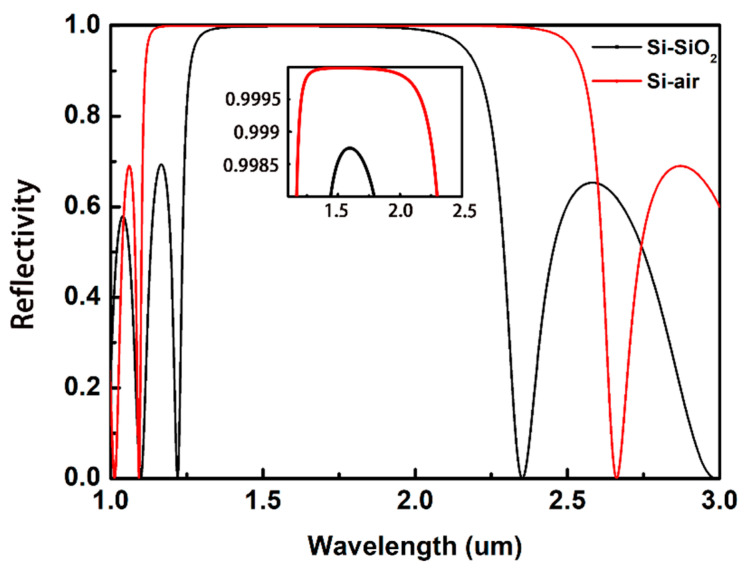
Stop bands of the designed DBRs. The inset shows the reflectivity of two DBRs around 1550 nm.

End-facet reflectivity is another critical parameter for a NW laser, which determines the quality of the cavity and has a strong influence on the lasing threshold [34]. The well-faceted NWs predominantly support axial Fabry-Perot waveguide modes [35]. The small size of NW causes significant mirror losses, resulting in a poor reflectivity of end facets. To enhance the end-facet reflectivity, asymmetric DBRs are placed on two ends of the NW. At one end, 5-pair silicon/air generates an ultrahigh reflectivity. While at the other hand, 5-pair silicon/silica yields a less high reflectivity to output laser power. The stop bands of asymmetric DBRs are shown in Figure 3. It can be seen that both the two DBRs generate a high reflectivity exceeding 99% in the telecommunication band, and the reflectivity of silicon/air pairs is higher than that of silicon/silica. 

## 3. Results and Discussion

**Figure 4:**
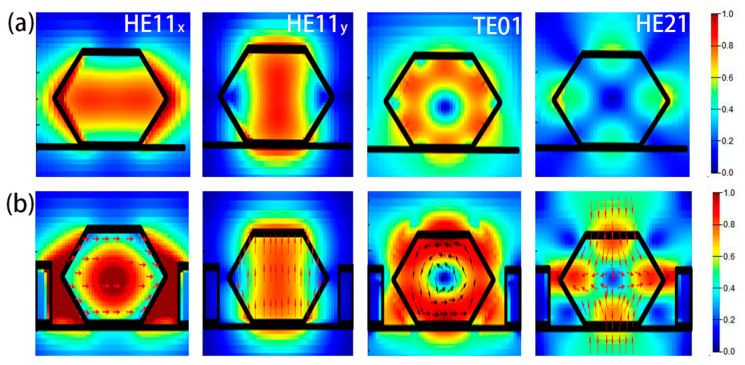
Normalized cross-sectional electric field distribution of HE11_x_, HE11_y_, TE01 and HE21 modes for (**a**) the pure NW on the silica platform, and (**b**) the PhC NW on the SOI substrate, respectively.

Due to the tight coupling of NW’s inherent F-P cavity, the PhC microcavity, and DBRs in the trench, the confinement of modes in the designed PhC-DBR NW laser should be much stronger than that in a pure NW laser. In order to verify our expectation, the normalized cross-sectional distribution of the first four guided modes in a pure NW and PhC NW is shown in Figure 4. Here, a structure with a PhC constant of 453 nm and NW radius of 200 nm is taken for example. Compared with the circular cross section, the hexagonal shape reduces the device symmetry, converting the fundamental mode HE11 into nonequivalent HE11_x_ and HE11_y_ modes. By comparing Figure 4a,b, it can be seen that more energy is confined inside the gain medium in the PhC NW laser compared with the pure NW for most modes, which means that the defect mode of PhC has a strong overlap with the gain medium. Besides, it is well established that a slow group velocity *v_g_* is easy to be achieved in defect modes in PhC [36,37,38,39], which leads to a much larger *Γ* for a PhC NW laser, compared with a pure NW. One exception is the HE11_y_ mode, whose intensity is weakened after introducing the PhC. According to the vector field of HE11_y_ depicted in Figure 4b, it is easy to find that HE11_y_ mode is y-polarized, which is perpendicular to the PhC slab and could not be confined in the trench efficiently. However, the surrounding silicon raises the environmental effective refractive index, which makes more photonic energy leaked from the NW.

**Figure 5:**
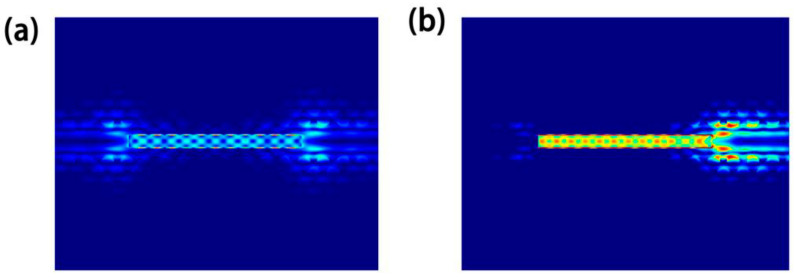
Normalized field intensity profiles of HE11_x_ mode for the (**a**) PhC NW and (**b**) PhC-DBR NW laser with a radius of 170 nm.

End-facet mirror losses rather than round-trip losses dominate the optical losses in NWs [34,40]. For all modes, the reflectivity reduces as the radius decreases, especially near the mode cut-off. So, a high end-facet reflectivity is critical for low-threshold miniaturized NW lasers. One the other hand, to maintain a high output power, one end should has a smaller reflectivity than the other. Figure 5a,b show the field intensity profiles of HE11_x_mode for a PhC NW and PhC-DBR NW laser, respectively. Much stronger optical resonances are observed in the PhC-DBR laser, which is attributed to the extra DBRs in the trench that significantly enhances the optical feedback. In addition, major optical power is extracted from one end due to the difference in reflectivity between DBRs at two ends.

**Figure 6:**
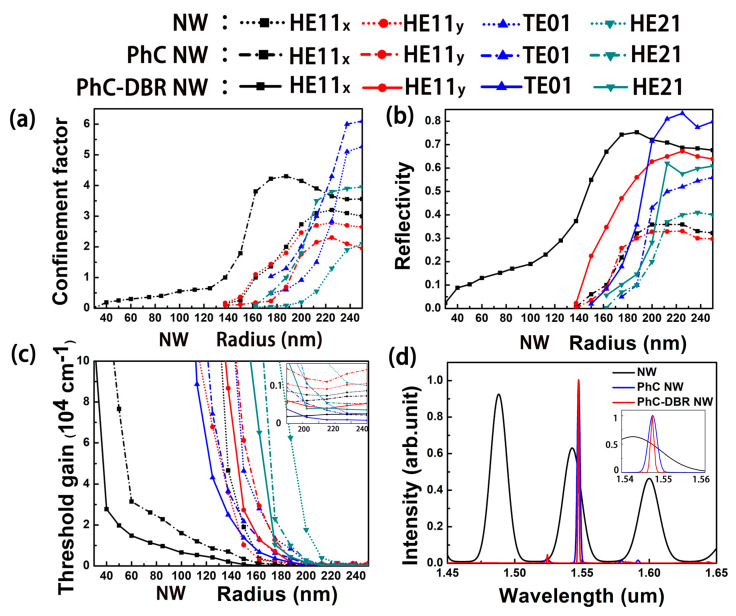
(**a**–**c**) Radius-dependent confinement factor, reflectivity, and threshold gain of NW lasers with different structures. (**d**) Longitudinal modes of HE11_x_ mode in three structures with a radius of 170 nm.

Threshold gain $\;g_{th}$ indicates the required gain per unit length for lasing, which is defined as [40,41],

$$g_{th}=\left(\frac{1}{\Gamma L}\ln\frac{1}{R_{1}R_{2}}\right)$$

where *Γ* is the confinement factor, L is the NW length, $R_{1}$ and $R_{2}$ are reflectivity at two end facets. The modal confinement factor is an indicator of coupling efficiency between the gain medium and resonant modes, which is given by [34,42],

Γ=cε0na(ω)∬active12|E|2dxdy∬∞12Re[(E×H∗)z⋅dxdy]

where E and H are the complex electric and magnetic fields of the NW mode, respectively. $\varepsilon_{0}$ is the vacuum permittivity, c is the vacuum light speed and $n_{a}$ is the refractive index of the gain medium (NW) at a particular frequency ω. The integration region of numerator is the gain medium, while the integral region of denominator is across the entire simulation area. Figure 6a shows the radius-dependent confinement factor of pure NW and PhC NW lasers, respectively. It can be seen that the confinement factor of most guided modes, except for mode HE11_y_, increases due to the strong subwavelength confinement of PhC microcavity. The PhC NW laser exhibits a maximum confinement of 4.3 for HE11_x_ mode at a radius of 188 nm, 1.34 times as high as the maximum value in a pure NW laser at a larger radius of 225 nm. After introducing DBRs, the end-facet reflectivity is significantly enhanced for all modes, as shown in Figure 6b. For HE11_x_ mode, the PhC-DBR NW laser exhibits a maximum reflectivity of 0.74 at a small radius of 175 nm, 2.1 times as high as the maximum value in the PhC NW laser at a larger radius of 200 nm. The increased confinement factor and enhanced reflectivity of the PhC-DBR NW laser result in a much lower threshold, as shown in Figure 6c. A minimum threshold gain of ~80 cm^−1^ is obtained in TE01, which is only one third that of the pure and PhC NW lasers. In addition, an ultra-small cut-off radius of ~40 nm is obtained in HE11_x_, which is 30% and 80% that of the pure and PhC NW lasers, respectively. The results suggest that the PhC microcavity and asymmetric DBRs significantly improve the device performance, and are promising to achieve low-threshold minimized lasers.

Semiconductor NW lasers usually exhibit multimode behavior due to the lack of mode selection capability. Generally speaking, the spectra typically exhibits multiple peaks with discrete frequencies, which are corresponding to transverse modes in a finite-length NW, as shown in Figure 6d. It is necessary to select the required longitudinal mode to realize a single-mode laser. The traditional way is to shorten the optical path of lasing cavity, that is, the length of NW, to expand the free spectral range (FSR) of the multimodes. However, the shortening of NW length leads to smaller gain medium, which increases the difficulty of lasing [1,34,43,44]. Here, single-mode lasing is achieved by using the bandgap of PhC and the conduction band of the line defect waveguide. The conduction band is not wide enough to support all longitudinal modes, which helps to select the required mode close to 1550 nm. As shown in Figure 6d, single-mode lasing is observed in both the PhC and PhC-DBR NW lasers. The quality factor Q of NW, PhC NW, and PhC-DBR NW is 59.5, 574.1, and 1192.3, respectively.

**Figure 7:**
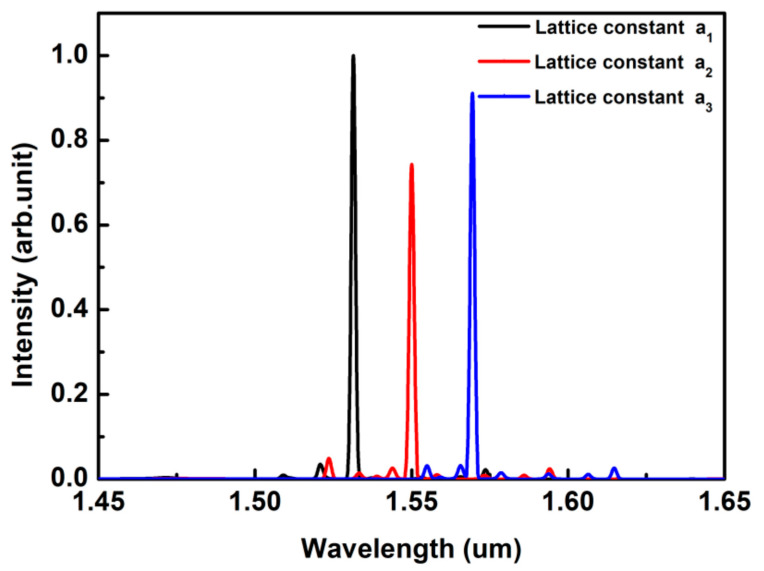
Output spectra of the multi-wavelength PhC-DBR NW laser array. All the longitudinal modes belong to HE11_x_. The radius is 120 nm.

As mentioned above, by engineering the lattice constant of the PhC, the lasing wavelength can be easily tuned. Here, we propose a multi-wavelength PhC-DBR NW laser array for on-chip wavelength division multiplexer (WDM) applications, as shown in Figure 1. The three NWs are same in material, dimension, and emitting spectra. By optimizing lattice constants of three PhCs, single-mode lasing is achieved at different wavelengths. The output spectra of three lasers are presented in Figure 7. The output spectra are centered at 1530 nm, 1550 nm, and 1570 nm, respectively, corresponding to three wavelengths of coarse WDM systems. The Q factors are 1456.4, 1233.2 and 1301.5, respectively. Moreover, all resonant modes show excellent single-mode behavior, with calculated high side-mode suppression ratio (SMSR) of 15 dB, 12 dB, and 14 dB, respectively.

## 4. Conclusions

In summary, we proposed and simulated a low-threshold miniaturized single-mode NW laser operating at telecommunication wavelengths. The device was composed of a single InGaAs NW, a PhC microcavity, and asymmetric distributed-Bragg-reflector mirrors. The mode characteristics and threshold properties were calculated using the FDTD method. The simulation results show that the PhC microcavity and DBR mirrors provide effective subwavelength confinement and strong optical feedback, respectively, leading to significantly enhanced confinement factor, end-facet reflectivity, and quality factor. A lowest threshold of ~80 cm^−1^ and ultra-small cut-off radius of ~40 nm are obtained, reduced by 67% and 70%, respectively, compared with a traditional NW laser. In addition, due to the photonic band gap effect, single-mode lasing is achieved with a high side-mode suppression ratio of >12 dB. By adjusting the lattice constant of the photonic crystal, the lasing wavelength could be effectively tuned, enabling the realization of on-chip laser array which can output multiple wavelengths. This work may open up new opportunities for the development of low-threshold miniaturized nanolasers and low-consumption high-density photonic integrated circuits.
